# Neural Functions of Matrix Metalloproteinases: Plasticity, Neurogenesis, and Disease

**DOI:** 10.1155/2012/789083

**Published:** 2012-04-10

**Authors:** Hiromi Fujioka, Yusuke Dairyo, Kei-ichiro Yasunaga, Kazuo Emoto

**Affiliations:** ^1^Department of Cell Biology, Osaka Bioscience Institute, 6-2-4 Furuedai, Suita, Osaka 565-0874, Japan; ^2^Graduate School of Biological Science, Nara Institute of Science and Technology, 8916-5 Takayama, Ikoma, Nara 630-0192, Japan

## Abstract

The brain changes in response to experience and altered environment. To do that, the nervous system often remodels the structures of neuronal circuits. This structural plasticity of the neuronal circuits appears to be controlled not only by intrinsic factors, but also by extrinsic mechanisms including modification of the extracellular matrix. Recent studies employing a range of animal models implicate that matrix metalloproteinases regulate multiple aspects of the neuronal development and remodeling in the brain. This paper aims to summarize recent advances of our knowledge on the neuronal functions of matrix metalloproteinases and discuss how they might relate in neuronal disease.

## 1. Introduction

In higher vertebrates, the space between neural cells in the brain is filled with material of the extracellular matrix (ECM). Both neurons and glial cells contribute to the production of the ECM components, and the ECM in turn mediates various structural and functional interactions between these cells [1, 2]. During early development, the ECM plays crucial roles in proliferation, migration and differentiation of neural cells. In the mature brain, the ECM undergoes a slow turnover and supports multiple physiological processes. In general, the mature ECM environment seems inhibitory for structural plasticity of neuronal circuits. For example, chondroitin sulfate proteoglycans appear to be one of inhibitory components in the ECM because their degradation by chondroitinase can reactivate ocular dominance plasticity [3]. It is thus likely that regulated proteolytic alteration of the ECM microenvironment should be required for the structural plasticity of neuronal circuits.

The ECM modifications in the nervous system are likely achieved by the concerted actions of several different proteases that are secreted by neurons and glial cells [4–6]. Among these proteinases, the matrix metalloproteinases (MMPs) family stands out as likely regulators of the neural plasticity. The mammalian central nervous system (CNS) contains over 10 different MMPs with detectable levels of transcripts or proteins [7, 8]. Studies of the temporal and spatial expression patterns of MMPs in the developing nervous system suggest that MMPs play important roles in neuronal development. In addition, expression of many MMPs has shown to change in response to injury or neurological disease [9]. Knockouts of particular MMPs significantly affect the injury and pathology, indicating that MMPs function as the crucial mediators of neuronal disease [10–12]. Interestingly, several MMP knockouts show deficits in learning and memory [10, 11]. Consistent with these notions, MMPs likely mediate the structural changes of dendritic spines as well as axon/dendrite structures in response to neuronal activity and in mental diseases [10, 11]. This paper considers potential roles for MMPs in neuronal development and plasticity, and discusses its alteration in injury and disease states.

## 2. MMPs in the Nervous System

**Figure 1:**
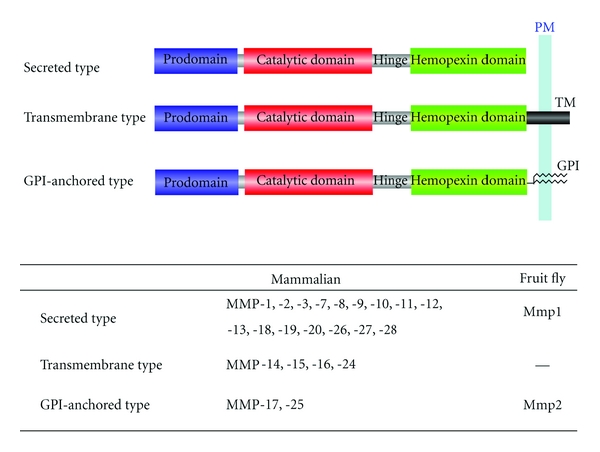
Domain structures of typical MMP family members in mammal and fruit fly. Hinge, hinge region; PM, plasma membrane; TM, transmembrane domain; GPI, glycosyl-phosphatidylinositol linker. The signal sequence located at the amino terminus of all prodomain is not shown.

Currently, 24 mammalian MMPs have been identified with distinct yet overlapping substrate specificities (Figure 1). Several MMPs are membrane anchored by transmembrane domains (MMP-14, -15, -16, -24) or by GPI links (MMP-17, -25). The expression of many MMPs has been detected in the nervous system and shown to change in response to injury and neurological disease [6–8]. Analysis of mRNA expression in the brain and spinal cord showed that MMP-2, -9, -11, -12, -13, -14, -15, and -24 are developmentally regulated, whereas mRNA levels of MMP-3, -7, and -10 remain unchanged throughout the neural development [6–8]. So far, two secreted types of MMPs, MMP-2, and MMP-9, are most frequently investigated MMPs in the brain because they are relatively easily detectable. MMP-2 is detected in various brain structures including astroglia and some pyramidal neurons in the cortex and Purkinje cells in the cerebellum, whereas MMP-9 is expressed in the hippocampus, cerebellum, and cortex, predominantly in neurons [6–8]. Levels of MMP-2 and MMP-9 are significantly elevated following ischemia, brain injury, and kainate treatment [6, 11], implying a role for MMP-2 and MMP-9 in remodeling of neural circuits in response to neural activity and brain damages.

In the fruit fly *Drosophila melanogaster*, there are only two MMP family members: Mmp1 and Mmp2 [13]. Mmp1 is a secreted proteinase, whereas Mmp2 is a GPI-anchored protein (Figure 1). Expression levels of Mmp1 and Mmp2 are dramatically elevated during metamorphosis in many tissues including the nervous system [13]. In agreement with the expression pattern, recent reports indicate that Mmp1 and Mmp2 play critical roles in remodeling of neural circuits in the peripheral nervous system [14, 15].

## 3. Secreted MMPs in Structural and Functional Plasticity of Synapses

Dendritic spine morphology and synaptic potentiation can both be dynamically modulated by proteins of the ECM and the cell-surface proteins with which they interact, which has long fueled the idea that regulated ECM remodeling has an important role in synaptic plasticity [16]. In the brain, MMPs are secreted by neurons and glial cells in an inactive (pro)form, and they become proteolytically active when several regulatory steps that result in removal of the propeptide are triggered in response to specific stimuli [5]. For example, studies have shown that in response to long-term potentiation (LTP) induction, MMP-9 rapidly becomes proteolytically active at perisynaptic sites and is essential for maintenance of LTP [17, 18]. Thus, perisynaptic MMP-9 proteolysis in response to LTP induction is likely critical for local remodeling of dendritic spine structures and functions necessary to support long-term synaptic plasticity [19–21].

LTP is a widely used cellular model for long-lasting synaptic plasticity that is thought to underlie learning and memory. MMP-9 knockout mice show behavioral impairments in hippocampus-dependent associative learning [11]. Furthermore, hippocampal slice cultures from MMP-9 knockout mice show impaired LTP, which can be restored by the application of recombinant MMP-9 [11]. In other studies, transient induction of hippocampal MMP-9 and MMP-3 levels was observed when animals were run in the Morris water maze, further supporting a role for MMPs in hippocampus-dependent learning [18]. Although the precise molecular mechanisms involved remain unclear, MMP-mediated synaptic potentiation can be inhibited in hippocampal slices by blocking integrin signaling, which suggests that MMP-9 regulates synaptic plasticity and LTP through integrins [11]. MMP-9 may exert its effects on integrins through the cleavage of laminin or other ECM components, exposing otherwise inaccessible RGD sites that can induce integrin signaling. It is also possible that MMP-9 may yield signaling functions through CD44 and integrins as shown in B cell lymphomas [22].

Alternatively, MMP-9 could regulate LTP by promoting lateral movement of glutamate receptors in excitatory synapses. Many synapses in the mature CNS are wrapped by a dense ECM, which likely acts as a spatial obstacle for glutamate receptors including N-methyl-D-aspartic acid- (NMDA-) type and alpha-amino-3-hydroxy-5-methyl-4-isoxazolepropionic-acid- (AMPA) type receptors. It is thus assumed that modification of the ECM components might facilitate structural and functional plasticity in synapses. Indeed, a recent study using a high-resolution fluorescent in situ zymography (ISZ) shows colocalization of MMP-9 with synaptic glutamate receptors [23]. Michaluk et al. [24] showed that addition of recombinant MMP-9 in hippocampal cultured neurons increased lateral diffusion of NMDA receptors but not AMPA receptors. They further showed that the motility change was not mediated by alteration in the net ECM structure nor by direct cleavage of NMDA receptors, but rather through modification of an integrin-dependent pathway [24]. On the other hand, Frischknecht et al. [25] indicated that enzymatic removal of the ECM increased extrasynaptic receptor diffusion and the exchange of synaptic AMPA receptors but not NMDA receptors. It is thus possible that membrane diffusion of AMPA and NMDA receptors is differently regulated by the ECM and MMPs in synapses, which may contribute to functional difference of AMPA and NMDA receptors in synaptic plasticity.

## 4. MMPs in Axon Regeneration

Several lines of evidence point toward a potential role of MMPs in axonal regeneration following an injury. First, expression of MMPs has been shown to correspond with periods of recovery [26]. In regenerating sciatic nerve fibers, MMP-9 expression is colocalized with phosphorylated neurofilament M, a marker for regenerative elongation [27]. Furthermore, the phosphorylated neurofilament M is also induced by MMP-9 treatment and inhibited by an anti-MMP-9 antibody treatment [27]. Regenerating axons also show immunoreactivity for MMP-2 and MMP-3 [28].

A second line of evidence comes from studies of their application to nonpermissive substrates. When primary dorsal root ganglion (DRG) neurons are grown on cryostat sections of normal adult nerves, neurite outgrowth is poor as the adult nerves constitute an unfavorable substrate. However, when the nerve sections are treated with MMP-2, which degrades inhibitory chondroitin sulfate proteoglycans (CSPGs) to expose permissive laminin, neurite outgrowth from DRG neurons is promoted [29]. In a chronically denervated distal tibial nerve segments, successful regeneration of axons is facilitated by supernatant from a neural stem cell line containing large quantities of secreted MMP-2, which likely degrades the CSPGs in the chronically denerved nerve sections [30].

Wallerian degeneration occurs after injury and involves the breakdown of myelin and axons, and removal of degenerating nerve components. This clearance is necessary for eventual repair. MMP levels increase during Wallerian degeneration, and this correlation is functionally significant as Wallerian degeneration of a transected nerve is delayed by MMP inhibitors [31].

In summary, the data for MMPs in axonal regeneration is scant but still suggestive. More definitive evidence, perhaps in the form of application of MMPs to an area of injury *in vivo* to improve axonal regrowth, is required.

## 5. Membrane-Anchored MMPs Promote Dendrite Remodeling

The ECM exerts a strong influence on dendrite morphogenesis in cultured neurons, as the ECM can affect dendrite patterning in part through ECM-neurite adhesive contacts mediated by cell adhesion molecules such as integrins [32]. ECM-neurite interactions have also been implicated in regulating structural plasticity of dendrites *in vivo*. For instance, blockage of the integrin-ECM interaction in RGCs [33] or genetic ablation of the integrin-mediated signaling in adult cortical neurons [34] causes progressive retraction of dendritic branches.

The ECM modifications in the nervous system are likely achieved by the concerted actions of several different proteases that are secreted by neurons and glial cells [4–6]. Among these many proteases, MMPs stand out as likely regulators of the dendrite development and pathology since MMPs are dramatically upregulated in particular neurons of the developing brain and are often colocalized with dendrites [35, 36]. However, in large part due to issues of redundancy and compensation among over 20 vertebrate MMP family members, the *in vivo* role of MMPs in the nervous system remains to be established (Table 1).

**Figure 2:**
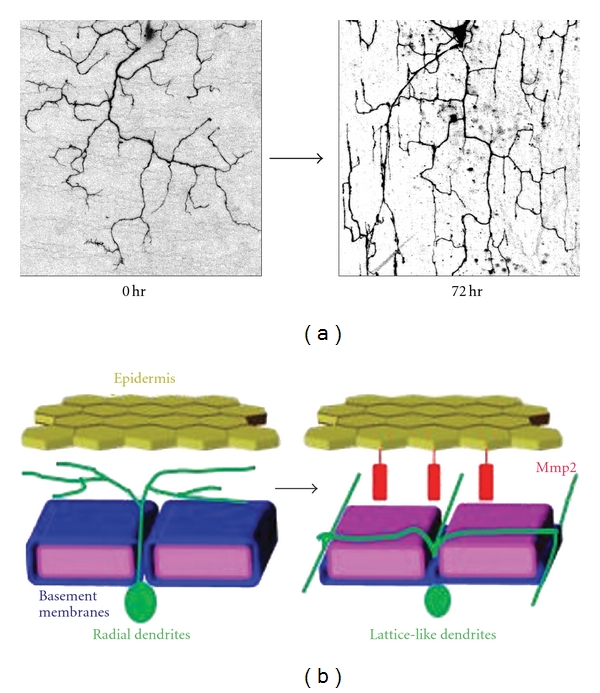
Dendrite reshaping in *Drosophila* sensory neurons is mediated by matrix metalloproteinases. (a) Dendritic pattern of a single class IV neurons in 0 hr and 72 hr posteclosion adults. (b) Dendrites of *Drosophila* sensory neurons are rapidly reshaped within 24 hours after eclosion. This dendrite reshaping is promoted by the matrix metalloproteinase Mmp2-mediated degradation of the basement membrane, suggesting that proteolytic alteration of the extracellular matrix plays a fundamental role in remodeling of dendritic structures during reorganization of neuronal circuits. This model is predominantly based on data from [15].

A recent study on *Drosophila* PNS neurons has provided *in vivo* evidence that ECM degradation facilitates dendrite remodeling [15]. During metamorphosis, the larval dendritic arbors of class IV sensory neurons are completely replaced with adult-specific processes as a result of extensive pruning and subsequent regeneration of dendritic arbors [14, 46]. Similar to their larval counterparts, dendrites of adult class IV neurons initially elaborate dendritic trees in a radial fashion and covered the whole body wall prior to eclosion. However, in contrast to what is observed during larval development [47–49], this radial arrangement of the dendritic arbor is rapidly rearranged to a lattice-like shape within 24 h after eclosion [15]. Time-lapse imaging revealed that this radial-to-lattice reshaping is largely due to rearrangement of the existing radial processes into a lattice-like pattern, rather than extensive pruning of the radially-arranged dendrites followed by regrowth of new arbors into a lattice pattern (Figure 2). Mutations in *Mmp2,* which encodes a GPI-anchored matrix metalloproteinase, block this radial-to-lattice reshaping of class IV dendrites without affecting other aspects of dendrite growth or development, and Mmp2 expression in epithelial cells adjacent to class IV dendrites is transiently increased at exactly the time when class IV dendrites undergo the radial-to-lattice reshaping. Therefore, epithelial Mmp2 promotes the dendrite reshaping through local modification of the basement membrane (BM) upon which class IV dendrites grow. These observations indicate that alteration of the ECM microenvironment might be a general mechanism for driving the structural plasticity of dendritic arbors *in vivo* (Figure 2).

In the mouse cerebellum, membrane-type 5 MMP (MT5-MMP; also named as MMP-24) is expressed in developing dendrites of Purkinje cells, implicating a role for MT5-MMP in dendrogenesis [35]. The precise roles of MT5-MMP in the cerebellum are not known. Since structural remodeling in dendrites as well as synapses is reported in developing Purkinje cells [50], it is of interest to examine if membrane-anchored MMPs including MT5-MMP may play a role in dendrite remodeling the mammalian nervous system.

## 6. Potential Roles of MMPs in Neurogenesis

MMPs have recently been considered to be involved in the neurogenic response of adult neural stem/progenitor cells. MMPs are expressed abundantly in neural stem cells isolated from the human CNS [42]. Brain injuries including ischemia enhance neurogenesis in neuroproliferative regions of the adult rodent brain such as the subventricular zone (SVZ) of the lateral ventricles and the subgranular zone (SGZ) of the dentate gyrus (DG) of the hippocampus [43]. Although most MMPs are expressed at very low levels in the adult CNS, mRNA expression of both MMP-9 and MMP-2 increased severalfold in neural progenitor cells of SVZ after ischemic insult in adult rats [44]. Similarly, upregulation of MMP-9 and MMP-2 in the SGZ of the dentate gyrus was compatible with the peak of postischemic neurogenesis in adult primate brains [45]. These observations suggest that MMPs could be an important component in neurogenesis-associated processes in postischemic brain hippocampus. Physiological significance of the MMP expressions in the neurogenic regions remains to be determined. One likely possibility is that, as shown in the developing brain, MMPs might promote proliferation, neurite extension, and migration of newly born neurons. Alternatively, MMPs might play a role in providing an optimal niche for neural stem/progenitor cells by modulating the ECM environment.

## 7. MMPs in Neuronal Injury and Disease

MMP expression levels are elevated after nervous system injury and in a number of neuronal pathologies. For example, MMP-9 expression is elevated shortly after ischemia and stroke [9]. Likewise, multiple MMPs including MMP-3, -7, -10, -11, -19, and -20 are immediately induced within 24 h of an acute insult such as spinal cord compression injury [51]. During the period of peak signs in experimental autoimmune encephalomyelitis (EAE), an animal model of multiple sclerosis and the transcripts encoding the majority of MMPs are elevated [52]. In epileptogenesis, serum MMP-9 levels and the ratio of MMP-9 to tissue inhibitor of metalloproteinase-1 are elevated in children with various febrile seizures and convulsive status epilepticus [53]. After seizure, MMP-9 mRNA is transported to dendrites and synapses in the hippocampal DG of kainic acid-treated rats [54]. Wilczynski et al. showed that the sensitivity to PTZ epileptogenesis was decreased in MMP-9 knockout mice but is increased in transgenic rats overexpressing MMP-9 [41].

The increase of many MMPs in CNS pathology raises the question of which of these enzymes are important for the pathophysiological process. The alleviation of disease pathology in response to MMP inhibitors shows that the net effect of the expression of MMPs in CNS injury is detrimental. In this regard, MMP-9 null mice have been found to be less afflicted than wild-type mice by EAE and stroke, whereas MMP-9 and MMP-12 null mice recover better from spinal cord injury [55, 56]. MMP-2 null mice have been found to be more suspective to EAE, which have been attributed to compensatory increase in MMP-9 in these animals [57]. In the mouse model of EAE, MMP-9 promotes development and progression of the disease whereas MMP-12 has a role in its resolution [58], suggesting that different MMPs might have distinct roles in EAE development. Indeed, double MMP-2 and -9 knockout mice are resistant to EAE development [59], indicating that combined MMP-2 and -9 activities are crucial EAE.

In addition to the brain injury and diseases, MMPs are implicated in drug addiction. The MMP inhibitors often suppressed acquisition of cocain-induced conditioned place preference (CPP) [60]. In consistent with the inhibitor studies, MMP protein levels in the brain were changed in cocain abusers as well as cocain-treated animals [61]. The role of MMPs in drug addiction was further confirmed by the findings that MMP-2 and MMP-9 deficient mice displayed attenuated sensitization and cocain CPP [62, 63].

MMPs are recently shown to be involved in development of neuropathic pain, which is characterized by mechanical allodynia that is, painful responses to previously nonpainful mechanical stimuli [64]. In L5 spinal nerve ligation, MMP-9 shows a rapid and transient upregulation in the injured DRG sensory neurons, whereas MMP-2 shows a delayed response in DRG satellite cells and spinal astrocytes. Local inhibition of MMP-9 by an intrathecal route inhibits the early phase of neuropathic pain, whereas inhibition of MMP-2 suppresses the late phase of neuropathic pain. These results suggest that distinct MMPs play different roles in early and late-phase development of neuropathic pain and that MMP inhibitors may provide a therapeutic approach for the treatment of neuropathic pain.

A recent report suggests a possible role of MMPs in Huntington's disease (HD) pathogenesis [65]. HD, the most frequent of a group of nine inherited neurodegenerative polyglutamine disorders, is caused by an expanded CAG triplet repeat in exon 1 of the huntingtin gene that encodes a stretch of polyglutamine (polyQ) residues close to the N-terminus of the huntingtin (Htt) protein. Htt is known to be cleaved by various proteases including Caspases and calpains, and inhibition of the mutant Htt proteolysis reduces neurotoxicity, indicating an important role for Htt proteolysis in HD pathogenesis. Miller et al. examined Htt proteolytic processing by screening for enzymes that cleave mutant Htt and found that MMP-10 is responsible for the Htt proteolysis to produce small N-terminal toxic fragments. A nonpeptidic inhibitor of MMPs NNHG, and two known endogenous MMPs inhibitors, TIMP1 and TIMP3, blocked Htt-mediated toxicity and produced beneficial therapeutic effects, providing new insights into Htt proteolysis and its potential as a therapeutic target.

## 8. Future Perspectives

This paper highlights the diversity and importance of MMPs in neuronal development, plasticity, and maintenance of neuronal health. Many questions await further studies, including which member or members of the MMPs are important in a particular condition or pathological state, how they achieve their effects, and what roles each MMP has in the overall scheme. MMPs are also implicated in diseases of the nervous system, and it is important to target their activity for therapy. The study of MMP functions should open up new vistas in the nervous system physiology and pathology.

## Figures and Tables

**Table 1:** MMP family proteins in neural circuit remodeling.

	Mammalian	Fruit fly
Synapse remodeling	MMP-3, -7, -9, -24 [11, 16–25]	No reports available
Axon regeneration	MMP-2, -3, -9, [27–31, 37]	Mmp1/2 [38, 39]
Dendrite remodeling	MMP-2, -9, -24 [40, 41]	Mmp1/2 [14, 15]
Neurogenesis	MMP-2, -9 [42–45]	No reports available
